# Remote sensing measurements of sea surface temperature as an indicator of *Vibrio parahaemolyticus* in oyster meat and human illnesses

**DOI:** 10.1186/s12940-017-0301-x

**Published:** 2017-08-31

**Authors:** Stephanie Konrad, Peggy Paduraru, Pablo Romero-Barrios, Sarah B. Henderson, Eleni Galanis

**Affiliations:** 1 Canadian Field Epidemiology Program, Public Health Agency of Canada, 130 Colonnade Road, Ottawa, Ontario K1A 0K9 Canada; 2 BC Centre for Disease Control, 655 West 12 Avenue, Vancouver, British Columbia V5Z 4R4 Canada; 3 School of Population and Public Health, University of British Columbia, 2206 East Mall, Vancouver, British Columbia V6T 1Z3 Canada

**Keywords:** *Vibrio parahaemolyticus*, Oysters, Illness, Sea surface temperature, Threshold model, Environmental factors, Remote sensing

## Abstract

**Background:**

*Vibrio parahaemolyticus (Vp)* is a naturally occurring bacterium found in marine environments worldwide. It can cause gastrointestinal illness in humans, primarily through raw oyster consumption. Water temperatures, and potentially other environmental factors, play an important role in the growth and proliferation of *Vp* in the environment. Quantifying the relationships between environmental variables and indicators or incidence of *Vp* illness is valuable for public health surveillance to inform and enable suitable preventative measures. This study aimed to assess the relationship between environmental parameters and *Vp* in British Columbia (BC), Canada.

**Methods:**

The study used *Vp* counts in oyster meat from 2002-2015 and laboratory confirmed *Vp* illnesses from 2011-2015 for the province of BC. The data were matched to environmental parameters from publicly available sources, including remote sensing measurements of nighttime sea surface temperature (SST) obtained from satellite readings at a spatial resolution of 1 km. Using three separate models, this paper assessed the relationship between (1) daily SST and *Vp* counts in oyster meat, (2) weekly mean *Vp* counts in oysters and weekly *Vp* illnesses, and (3) weekly mean SST and weekly *Vp* illnesses. The effects of salinity and chlorophyll a were also evaluated. Linear regression was used to quantify the relationship between SST and *Vp*, and piecewise regression was used to identify SST thresholds of concern.

**Results:**

A total of 2327 oyster samples and 293 laboratory confirmed illnesses were included. In model 1, both SST and salinity were significant predictors of log(*Vp*) counts in oyster meat. In model 2, the mean log(*Vp*) count in oyster meat was a significant predictor of *Vp* illnesses. In model 3, weekly mean SST was a significant predictor of weekly *Vp* illnesses. The piecewise regression models identified a SST threshold of approximately 14^o^C for both model 1 and 3, indicating increased risk of *Vp* in oyster meat and *Vp* illnesses at higher temperatures.

**Conclusion:**

Monitoring of SST, particularly through readily accessible remote sensing data, could serve as a warning signal for *Vp* and help inform the introduction and cessation of preventative or control measures.

## Background

*Vibrio parahaemolyticus* (*Vp*) is a naturally occurring bacterium found in marine, estuarine, and freshwater environments throughout the world. *Vp* is one of approximately a dozen *Vibrio* species known to cause illness in humans, including *Vibrio vulnificus* and *Vibrio cholerae*, and is an important cause of seafood-associated gastroenteritis globally [1]. On the Pacific coast of North America, which includes the study area of British Columbia (BC), Canada, the incidence of *Vp* illnesses has continually increased since the mid-2000s, though the underlying causes remain unclear [2–4]. Notable *Vp* outbreaks affecting the Pacific Northwest occurred in 1997 and 2015 [5, 6]. Both of these outbreaks were associated with raw oyster consumption and above average sea water temperatures [5, 7].

Raw shellfish, particularly oysters, are the most common foodborne source of *Vp* in BC. Oysters are filter feeders and thus accumulate *Vp* when present in the water. Other sources for infection include direct ingestion of contaminated water, open wound exposure and ear infections [2, 3]. The gastrointestinal symptoms associated with *Vp* infection are generally self-limited, moderate in severity, and lasting 1-7 days. Systemic infection and death rarely occur [8].

Many environmental factors play an important role in the growth of *Vp*, the subsequent availability of *Vp* for accumulation by shellfish and, finally, *Vp* infection in humans who consume contaminated shellfish*.* While the specific relationship varies by region, increasing water temperatures are generally associated with increasing prevalence of environmental *Vp* [9–18] and incidence of *Vp* illnesses [19, 20]. In fact, ocean warming has been noted as an emerging risk for all *Vibrio* infections [21]. The relationship between *Vp* and other environmental factors, including salinity, turbidity, and phytoplankton may also play an important role, though studies have reported inconsistent results [9, 10, 12–17]. These factors are thought to affect the availability and growth of *Vp* by releasing the bacterium and other nutrients from the sediments (turbidity), enriching *Vp* (phytoplankton), and providing the optimal environmental conditions to enable growth (salinity) [13, 15, 22]. There are likely interdependencies between these variables that are driven by unique local conditions [11]. Nevertheless, water temperature has been the most important environmental predictor, explaining 43-70% of the variability seen in *Vp* counts [10, 17, 18].

Quantifying the relationship between environmental variables and *Vp* indicators or incidence of human disease is valuable for public health surveillance to inform and enable suitable preventative or control measures [23]. For example, research in East Africa showed that environmental factors could predict *Vibrio cholerae* outbreaks at least one month in advance, allowing for preventative measures such as vaccination [24]. Another study in the US has evaluated and proposed monitoring satellite-based remote sensing environmental data for predicting the incidence and risk of *Vp* in oyster meat around the Gulf of Mexico [14].

**Fig. 1:**
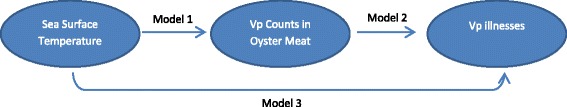
Summary of the three models used to assess *Vp* risk. Model 1 used data from 2002-2015, while models 2 and 3 were limited to data from 2011-2015

The recent outbreak of *Vp* in BC spurred interest in exploring an early warning system to inform on the risk of *Vp*. Given the potential utility for environmental surveillance through remote sensing data and the emerging risk of warming water temperatures, this study aimed to assess the relationship between environmental parameters and *Vp* indicators in BC. The impacts of environmental parameters, including sea surface temperature (SST), are not well understood in BC*.* Compared with other locations, BC coastal waters are generally cooler, receive more fresh water in the form of precipitation and discharge, and are inhabited by a more diverse flora and fauna [25]. Using remote sensing measurements of SST, *Vp* counts in oyster meat, and laboratory-confirmed cases of *Vp* illnesses we assessed (1) the ability of SST to predict *Vp* in oyster meat, (2) the ability of *Vp* in oyster meat to predict *Vp* illnesses, and (3) the ability of SST to predict *Vp* illnesses (Fig. 1). The overall strength and consistency of these relationships can provide evidence to support the utility of routine SST surveillance along the BC coast as an indicator of *Vp* risk.

## Methods

### Study area

**Fig. 2:**
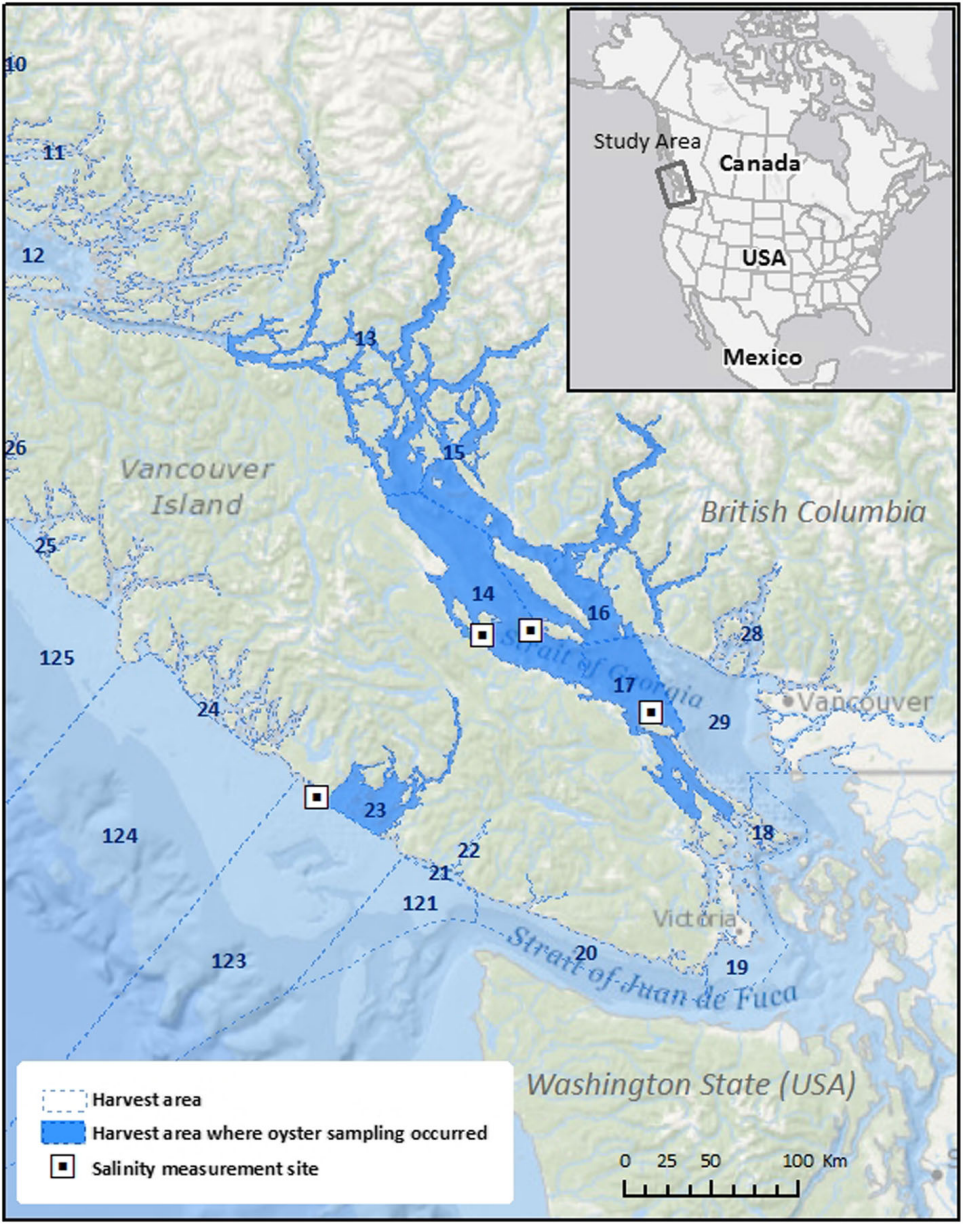
Harvesting areas of British Columbia with locations of the oyster sampling and salinity data sites

The province of BC is located on the west coast of Canada, with a vast coastline along the mainland and around Vancouver Island (Fig. 2). These coastal waters support a vibrant oyster farming industry, which includes intertidal and suspended culture farming practices. Only the Pacific Oyster (*Crassostrea gigas*) is grown in BC. BC’s oysters account for approximately 60% of the Canadian oyster production. This equates to 5,600 tonnes of oysters annually, with a 2013 market value of $27.3 million [26]. The 2015 population of BC was approximately 4.7 million residents, the majority of which live in close proximity to the coast in greater Vancouver (2.3 million), on Vancouver Island (767,000), or elsewhere (186,000) [27].

### Environmental data

Estimates of SST (in ^o^C) were obtained for the 2002-2015 period from the satellite-based Multiscale Ultrahigh Resolution (MUR) product computed by the National Aeronautics and Space Administration (NASA). The SST estimates are available at a spatial resolution of 1 km and based on composite daily values computed with nighttime observations from multiple satellites using thermal-infrared and microwave wavelength instruments. Estimates are validated against in-situ observations [28]. Chlorophyll a measurements (in mg/m^3^) from 2003-2015 were obtained from the daytime overpasses of the NASA Aqua satellite. Chlorophyll a is measured using the Moderate Resolution Imaging Spectroradiometer (MODIS) instrument, and the data are available at a spatial resolution of 4.6 km [29]. Salinity data (in practical salinity units, or psu) from 2002-2015 were obtained from the Fisheries and Oceans Canada BC Lighthouse database [30]. These daily measurements are made manually at high tide with a Conductivity Temperature and Depth probe [31]. Data were available from four sites within the provincial shellfish growing regions (Fig. 2) [32].

### *Vp* measurements in oyster meat

Historical total *Vp* counts in oyster meat were provided by the Canadian Food Inspection Agency (2002-2013) and three shellfish producers (2004-2015), randomly categorized as data sources A, B, C, and D. Data were available for the months of April-October, and each observation included the collection date and either the geographic coordinates of the sampled bed or the name of the harvest area. Harvest areas are commercial aquaculture regions designated by Fisheries and Oceans Canada. Six different harvest areas and 39 distinct sets of coordinates were represented (Fig. 2). The *Vp* counts were measured using the most probable number (MPN) technique, a semi-quantitative method that estimates bacterial concentrations using serial dilutions, and were expressed in units of MPN/g [33]. Briefly, samples assessed with the MPN technique are prepared in ten-fold dilution series that are typically inoculated into broth in three-tube series at increasing dilution rates. Following incubation for 16-18 hours at 35°C, the tubes are examined for turbidity, which indicates growth. The presence of *Vp* is then confirmed using plating into selective media and using biochemical tests [34] or the polymerase chain reaction (PCR) method [35]. The number of positive tubes is converted into *Vp* counts using MPN tables. Over the study period, different limits of detection (<3 or <30 MPN/g) were used. To provide a numerical value for those results <3 MPN/g, a value of 1.5 MPN/g was assigned. For those observations with a value <30 MPN/g, an integer value between 3 and 30 MPN/g was randomly sampled from the distribution of available measurements within this range. The *Vp* counts were log-normally distributed, and all regression analyses were conducted with values transformed by taking the natural logarithm.

### *Vp* illnesses in humans

Laboratory confirmed illnesses from 2011-2015 were obtained from the provincial electronic public health information system. Because *Vp* illnesses are relatively rare, the daily data were aggregated to weekly counts for the regression analyses. Infection with *Vp* is a reportable disease in BC, and all stool samples are routinely cultured for *Vp* at all clinical laboratories across the province. Any individual with confirmed *Vp* illness is interviewed using a standardized questionnaire [36]. If shellfish consumption is the likely source of the illness, information about the source restaurant, facility, or retail location is gathered from the case. This information allows public health authorities to request the associated shellfish tags, which are kept with the product as it moves through the supply chain. Shellfish tags include information about the producer, the harvest area, and the harvest date.

There is a delay between the time at which an oyster is harvested and the time at which any illness caused by that oyster is reported to public health authorities. To adequately match the temporal environmental variables with the *Vp* illnesses in models 2 and 3, data from shellfish tags were used to estimate this delay. First we identified a subset of *Vp* illnesses in 2014-2015 for which shellfish tags had been identified. Then we calculated the median difference (in days) between the date of oyster harvest and date on which the illness was reported to public health authorities. Some illness reports were associated with multiple tags (e.g. one individual consumed a variety of oysters). In these cases the mean delay time was used in the calculation of the overall median.

### Ability of environmental variables to predict *Vp* counts in oyster meat (Model 1)

The SST, salinity, and chlorophyll a values were spatially matched to the location of each sample of *Vp* in oyster meat based on its collection date from April-October of 2002-2015. For the SST variable the location was taken as the geographic coordinates of the sample, where available, or the centroid of the harvest area. Because chlorophyll a and salinity data were less spatially resolved than the SST data, the location was taken as the centroid of the harvest area for these variables. The daily chlorophyll a value was calculated as the average of the values found within a 20 km radius of the centroid location to account for missing measurements. The daily salinity values for the four sites were matched to each harvest area based on proximity.

Colinearity between the SST, salinity, and chlorophyll a was assessed using Spearman’s rank analyses. The effect of SST was considered on the day of sampling (SST_0_), lagged by one to three days (SST_1_-SST_3_), averaged over three or four days (SST_0-2_ and SST_0-3_), or based on the maximum daily value over the same lag periods (MaxSST_0-2_, MaxSST_0-3_). Each SST metric was tested in linear models with the log(*Vp*) count, and the metric with the highest coefficient of determination (R^2^) was chosen for all further analyses. Linear regression was used to assess the relationship between the chosen SST metric, salinity, chlorophyll a, and log(*Vp*). The effects of the chosen SST metric, salinity, and chlorophyll a were then assessed in a multivariable linear regression model. All models were adjusted for data source, harvest area, and year and month of collection to assess the effects of these potential confounders. The final model was constructed using a forward stepwise approach, and the contribution of each variable to the model was evaluated based on the change in the adjusted R^2^. Finally, a piecewise regression was applied to the final model, to estimate the threshold value of the chosen SST metric at which the values of log(*Vp*) began to significantly increase. Final models were also restricted to the 2011-2015 period for comparison with results from the *Vp* illness data.

### Ability of *Vp* counts in oyster meat to predict *Vp* illnesses (Model 2)

Simple linear regression was used to estimate the association between *Vp* in oyster meat and *Vp* illnesses. Weekly counts of *Vp* illness from April-October of 2011-2015 were regressed against weekly average log(*Vp)* counts in oyster meat from all harvest areas. The *Vp* illness data were offset by the reporting delay to temporally match *Vp* counts, as described above.

### Ability of environmental variables to predict *Vp* illnesses (Model 3)

To calculate a weekly SST value that was representative of oyster growing areas in BC, we identified all the harvest sites (i.e. land files, the most resolved geographical unit of a harvest area) that produced oysters for raw consumption from 2011-2015 [37]. The same day SST values (SST_0_) at the centroid of these sites were averaged to produce a single daily SST value for all harvest sites, which was used to calculate the weekly average SST. The effect of weekly SST on weekly *Vp* illnesses was assessed using simple linear regression and limited to the same April-October time period used in models 1 and 2. A piecewise regression was subsequently applied for the entire year to assess the SST threshold at which at which the weekly *Vp* illness counts began to significantly increase. The *Vp* illness data were offset by the reporting delay to temporally match weekly STT data, as described above. Repeat analyses were conducted on the subset of *Vp* illnesses with known consumption of raw oysters.

All analyses were conducted using STATA 13.0 and R version 3.2.1 (R Development Core Team, 2014).

## Results

**Fig. 3:**
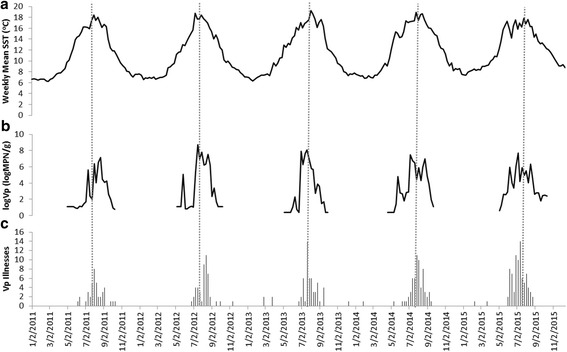
Time series of weekly SST, *Vp* counts in oyster meat, and *Vp* illnesses. The weekly SST data (a) were available all year. The Vp counts in oyster meat (b) were only available from April to October. *Vp* illness counts (c) are presented two weeks prior to reporting week to account for reporting delay. Dashed vertical lines represent mid-July of each year

A total of 2327 oyster samples were collected from 2002-2015. The *Vp* counts ranged from below the limit of detection to 11,000 MPN/g, with an arithmetic mean value of 317 MPN/g, a median of 7.2 MPN/g, and a geometric mean of 14.12 MPN/g, which is the antilog of the mean of all the log(*Vp*) values (Table 1). There was a strong seasonal trend, with the highest *Vp* count values generally in July and August when the SST measurements were highest (Fig. 3). The different SST metrics described between 13% and 24% of the variability in the log(*Vp*) counts, and all models that included the same-day value (SST_0_, SST_0-2_, SST_0-3_, MaxSST_0-2_, and MaxSST_0-3_) outperformed models that did not (SST_1_-SST_3_). The same day metric (SST_0_) was best fitted to the data (R^2^ = 0.24, p < 0.001), and thus was used for all further analyses.

**Table 1:** Descriptive statistics for *Vp* counts in oyster meat and the potentially predictive environmental variables

Variable	N	Interquartile Range	Mean	Median	Standard Deviation
*Vp* count (MPN/g)	2327	3 – 43	317	7.2	1419
log(*Vp*) count (log MPN/g)	2327	1.1 – 3.7	2.7	2.0	2.2
Sea surface temperature (^o^C)	2153	14.3 – 17.1	15.6	15.8	2.0
Salinity (psu)	1980	26.2 – 28.4	27.2	27.4	2.3
Chlorophyll a (mg/m^3^)	840	4.7 – 35.8	22.4	15.2	21.4

**Fig. 4:**
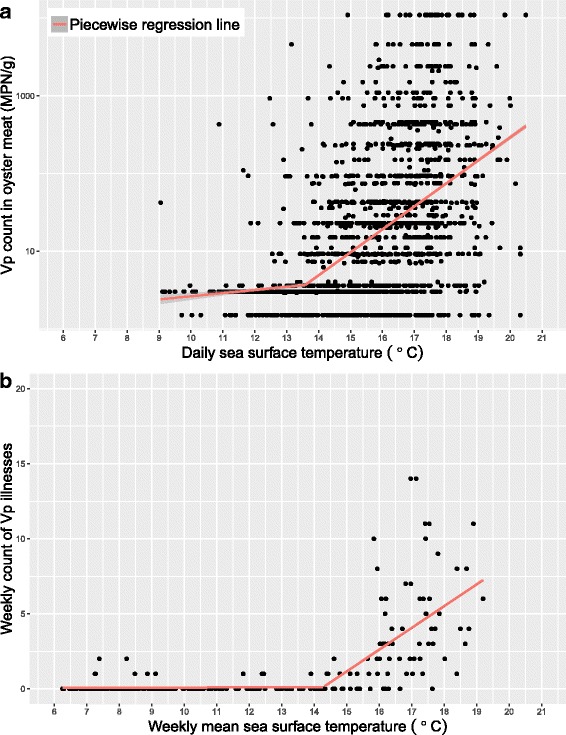
Scatterplot of Vp versus SST with fitted piecewise regression model. Vp counts in oyster meat (**a**) were available from April-October, 2002-2015. Laboratory confirmed *Vp* illnesses (**b**) include data from January-December, 2011-2015. Weekly sea surface temperature (SST) (b) was lagged by two weeks to accurately represent water temperatures at time of harvest, due to *Vp* illness reporting delay

No collinearity was found between SST_0_, salinity, and chlorophyll a. Simple linear regression models found a strong positive association between SST_0_ and log(*Vp*), with a 75% [95%CI: 65%, 84%] increase in *Vp* counts for each 1.0^o^C increase in SST_0_. There was a weak negative association between salinity and log(*Vp*), with an 8% [6%, 10%] decrease in *Vp* counts for each 1.0 psu increase in salinity. There was no association between chlorophyll a and log(*Vp*), and this variable was removed from further analyses (Table 2). The multiple linear regression model found that both SST_0_ and salinity were significant predictors of log(*Vp*), and that these relationships were not affected by inclusion of the harvest area, year, and month variables. However, adjusting the model for the data source increased the R^2^ value from 0.25 to 0.28, so this variable was included in the final model to account for potential variability in testing practices. The *Vp* counts from data source C were lower than those from data source A, while those from data source D were significantly higher. The piecewise linear regression model including the SST_0_, salinity, and data source variables identified a SST_0_ threshold of 13.6 ^o^C [12.9 ^o^C, 14.3 ^o^C] (Fig. 4). When not adjusted for the salinity and data source variables, the SST_0_ threshold from the piecewise model was still 13.6 ^o^C, but the confidence intervals were wider. When the data were restricted to the 2011-2015 period for comparison with model 3, the piecewise linear regression model including the SST_0_, salinity, and data source variables identified a SST_0_ threshold of 14.3 ^o^C [13.3 ^o^C, 15.4 ^o^C].

**Table 2:** Summary of linear regression analyses for variables predicting log(*Vp*) counts in oysters. Estimates in bold are significant at the 0.05 level, and the percent change in *Vp* counts is given only for significant estimates

Variable(s) in linear model	Coefficient [95%CI]	Change in *Vp* count per 1-unit increase [95%CI]*	Model R^2^
SST	**0.56 [0.51, 0.61]**	↑ 75% [67, 84] increase	0.24
Salinity	**-0.08 [-0.10, -0.06]**	↓ 8% [6, 10] decrease	0.04
Chlorophyll a	0.00 [-0.01, 0.01]	-	0.00
SST	**0.54 [0.50, 0.59]**	↑ 72% [65, 80] increase	0.25
Salinity	**-0.04 [-0.08, 0.00]**	↓ 4% [0, 8] decrease	
SST	**0.58 [0.53, 0.63]**	↑ 79% [70, 88] increase	0.28
Salinity	**-0.04 [-0.08, 0.00]**	↓ 4% [0, 8] decrease	
Data source**			
A	Reference		
B	-0.18 [-0.39, 0.15]	-	
C	**-1.64 [-2.11, -1.16]**	↓ 81% [69, 88] decrease	
D	**0.28 [0.00, 0.55]**	↑ 32% [0, 73] increase	

*Because analyses were conducted with log(*Vp*) counts, the change per each 1-unit increase is calculated by taking the antilog of the variable coefficient and taking difference from the baseline value of 1 (i.e. a coefficient of 0)

**Effect estimates reflect the change in Vp counts when compared with category A. Bolded coefficient represent statistically significant results at a p value of less than 0.05

There were 293 laboratory-confirmed *Vp* illnesses reported from 2011-2015. The *Vp* illnesses followed a seasonal trend consistent with that observed for *Vp* in oyster meat (Fig. 3). Exposure information was available for 179 of the 293 illnesses, of which 163 (91%) had consumed raw oysters. Forty-seven illnesses from 2014-2015 had accompanying shellfish tags and were used to estimate the reporting delay. The median delay between the harvesting and reporting date was 17 days, with an interquartile range of 8 days. Because the analyses were conducted with weekly data for models 2 and 3, this value was rounded down to 14 days. For example, the *Vp* illness count for the week of July 15-21, 2012 would be matched to the mean SST_0_ for the week of July 1-7, 2012 and the mean *Vp* count in oyster meat for the week of July 1-7, 2012.

In model 2, the simple linear regression between the weekly *Vp* illnesses and weekly mean *Vp* counts in oyster meat found that a log increase in the mean *Vp* count was associated with a 0.89 [0.70, 1.01] increase in the number of weekly *Vp* illnesses (R^*2*^=0.42). More simply put, each natural log increase in the province-wide average of *Vp* counts in oyster meat was associated with approximately one extra laboratory confirmed case of *Vp* illness two weeks later.

Seasonal peaks in *Vp* illnesses also paralleled mean SST_0_ patterns during the summer months (Fig. 3). In model 3, the simple linear regression found a 1.0 ^o^C increase in the weekly mean SST_0_ was associated with a 0.77 [0.57, 0.97] increase in the weekly number of *Vp* illnesses (R^2^=0.34). However, a scatterplot of the weekly mean SST_0_ and *Vp* illnesses showed no relationship at lower temperature until a particular point, indicating a that piecewise regression was more appropriate than simple linear regression for modeling the relationship. The piecewise model identified a weekly SST_0_ threshold of 14.3^o^C [13.6^o^C, 15.0^o^C], with an additional 1.45 [1.14, 1.77] cases of *Vp* illness for each 1.0 ^o^C increase over this value (R^2^=0.55) (Fig. 4). In the subset analyses restricted to cases with known raw oyster consumption, the weekly SST_0_ threshold was 14.2^o^C [12.1^o^C,15.3^o^C].

## Discussion

We found that remote sensing measurements of daily SST could be used to predict *Vp* counts in oyster meat samples. *Vp* testing of oysters*,* along with numerous regulations, exists to reduce health risks associated with consumption of shellfish. Even with concerted efforts to control *Vp* risk, we found that the weekly mean *Vp* counts in all tested oyster meat samples could be used to predict cases of *Vp* illness across the province two weeks after the samples were tested. Finally, we found that weekly mean SST at the estimated time of oyster harvest could be used to predict weekly cases of *Vp* illness two weeks later, when illnesses are reported. It follows that daily measurements of SST can provide a foundation for surveillance of *Vp* risk in commercial shellfish and the BC population.

Our findings are consistent with other studies in the Pacific Northwest, which have reported that increasing water temperatures are positively associated with increasing *Vp* concentrations in oysters [38–40]. Although the correlation is generally positive, Paranjpye et al. [41] found that the highest concentrations of *Vp* occurred approximately one month before the peak water temperatures were reached. Regardless, the authors found that *Vp* risk began to increase when temperatures reached the 12-15^o^C range, and that nutrient concentrations were also a significant predictor. One important consideration in BC and the Pacific Northwest is the practice of intertidal farming, which may complicate the relationship between SST and *Vp* concentrations. Air temperatures are generally warmer than water temperatures during the summer months, meaning that intertidal oysters may be warmer than the SST estimates suggest when tides are low [42]. Future studies should endeavour to stratify oyster meat samples by the farming practices used to cultivate them.

Despite potential confounding by farming practices, we found that SST was the most significant environmental predictor of *Vp* in oyster meat. We also found that sea surface salinity was negatively correlated with *Vp* in oyster meat, though the relationship was weak and it did not modify the effect of SST in multiple linear regression. The US Food and Drug Administration’s quantitative risk assessment model found a significant quadratic relationship between salinity and *Vp* in regions with a wider range in salinity, but not in the Pacific Northwest, where the range was smaller compared with other areas assessed [38]. Overall, the reported relationship between salinity and *Vp* seems to vary by geography, which may be due to the different ranges in salinity, different tolerances of local *Vp* populations, or the non-linear effects observed in some studies [11, 14, 18]. Finally, we found that chlorophyll a was not associated with *Vp* in oysters, which is consistent with one study [41], though not another [9]. This relationship has been noted with other *Vibrio spp*. [15, 16]. Regardless, for the linear regression models, we found that SST alone described 24% of the *Vp* variability in oyster meat, compared with the 28% described by the best three-variable model. Likewise, SST alone was able to describe 34% of the variability in *Vp* illnesses, suggesting that it is a valuable indicator for surveillance programs to monitor.

For the 2011-2015 period the daily SST threshold at which the risk of *Vp* in oysters began to increase was 14.3^o^C [13.3^o^C, 15.4^o^C]. The weekly SST threshold for the risk of *Vp* illnesses was almost identical at 14.3^o^C [13.6^o^C, 15.0^o^C] for the complete dataset and at 14.2^o^C [12.1^o^C,15.3^o^C] when restricted to cases with known oyster consumption. We have identified these thresholds using piecewise regression, which is a common approach in the field of occupational health [43]. Although other studies have not used the same methods to identify specific thresholds, they have reported that daytime water temperatures exceeding 15^o^C were associated with increased *Vp* counts in oysters [40] and *Vp* illnesses in the Pacific Northwest [3, 44]. Based on our comparisons with daytime SST manually measured in BC, the daytime values are typically 1^o^C warmer than the nighttime SST measurements we used for these analyses (not shown). Another study from the US Atlantic coast reported that *Vp* was released from sediments and detectable in those waters at 14^o^C [45]. Other thresholds reported in the literature include 17^o^C for the Georgian Coast of the Black Sea [10] to 18^o^C in France [12]. These differences may be due to differing methods and/or differing contexts, including the *Vp* strains, oyster species, and environmental conditions [46]. Together these studies highlight the need to carefully quantify the relationship between SST and *Vp* in all shellfish harvesting areas. The methods we describe here could easily be broadly applied.

There are some important limitations to these analyses. First, post-harvest practices can affect the growth or purging of *Vp* in contaminated oysters, which will subsequently affect the number of illnesses. For example, an oyster may have low concentrations at the harvest site, but temperature abuse along the supply chain can lead to rapid *Vp* growth [38] and we could not asses these intermediary factors. However, we don’t have evidence that these practices differed significantly during the study period, with the possible exception of behavioural changes during the 2015 outbreak.

Second, our models explain some, but not all, of the variability in the number of *Vp* illnesses or *Vp* counts in oysters. Other environmental parameters, such as water turbidity, have been significant predictors of *Vp* in other geographic areas [12], but such data were not available in BC. Furthermore, the chlorophyll a and salinity data were only available at very low resolutions, which may have affected the validity of the relationships we were able to report. The value of these models would therefore benefit from the inclusion of additional predictors such as these environmental parameters or information about harvesting practices.

Third, we only had measures of total *Vp* and not specifically pathogenic strains of *Vp*, although the latter also show similar seasonal trends and relationships with temperature [15, 22]. Fourth, our models for *Vp* in oyster meat and *Vp* illnesses used different time scales due to the limited number of laboratory-confirmed cases. Moreover, the different time scales serve two different purposes. For public health, daily fluctuations in temperature limit the usefulness of this time scale to indicate the onset of a period of higher risk, whereas the weekly mean is more stable. However, for the shellfish industry, a daily threshold is more suited to immediate risk mitigation. Regardless, both approaches suggested that nighttime SST of greater than 14^o^C at the time of harvest was associated with increased *Vp* risk. Caution, however, is warranted when applying this threshold of 14^o^C if other methods to measure temperature are used, such as in situ measurements or data available from measuring stations, as there will be differences in temperature between those local measurements and nighttime SST readings at a relatively coarse spatial resolution of 1 km. The same applies for oysters harvested from deep water, which may have considerably lower temperatures at lower depths.

This study highlights the utility of the validated MUR SST product from NASA. These remote sensing data are freely and publicly available in near-real time at high spatial and temporal resolutions. Ours is one of the first studies to assess the relationship between SST and *Vp* using remote sensing data [14], though others have recognized the value of this tool for monitoring environmental risk factors [23]. Numerous other studies, however, have used routine monitoring of remote sensing data to inform on the risk of *Vibrio spp.* [21] with progress towards early warning systems in Europe and Chile [47, 48]. Remote sensing data allows for low-resource near-real-time monitoring of SST to inform on the risk of *Vp*.

The BC Centre for Disease Control (BCCDC) implemented monitoring of the MUR SST data in 2016, following a large outbreak of *Vp* illness in 2015. The weekly average SST was compared with the 14.3^o^C threshold identified here to (1) indicate the start and end of *Vp* season, (2) initiate public health messaging around the risk of *Vp* from raw oyster consumption, and (3) inform the cessation of public health interventions. During the 2016 season SST reached the 14.3^o^C threshold four weeks before any *Vp* illnesses were reported. After accounting for the mean two-week reporting delay, this was two weeks longer than expected. However, the total number of *Vp* illnesses in 2016 was below the five year historic level. The low count could be partially attributable to the numerous industry, regulatory, and public health interventions that were implemented following the 2015 outbreak. For example, new testing guidelines were implemented by the Canadian Food Inspection Agency [49]. These actions would attenuate the effect of SST on *Vp* illnesses, and may serve to increase the threshold value. Even so, monitoring SST remains valuable for indicating the potential of increased risk and for encouraging and promoting control measures to reduce illnesses.

In addition to internal monitoring of weekly SST averages, the BCCDC began posting daily SST temperatures at 13 shellfish harvesting areas on its website. While the MUR SST data are freely and publicly available, it does require considerable expertise to download, process, and extract the data for human interpretation. The BCCDC has automated these steps such that a map on the website is updated every morning, and each harvest site displays SST measurements for the past 14 days. This format provides easily accessible data to a range of stakeholders, including shellfish farmers, processors and public health professionals with the intent to assist in risk assessment and management [50].

Our study contributes to a growing literature on the relationship between environmental variables and *Vp* risk, and it addresses specific gaps for the BC geographic area. We included data spanning more than ten years, and we confirmed the associations between (1) SST and *Vp* in oysters, (2) *Vp* in oysters and *Vp* illnesses, and (3) SST and *Vp* illnesses. The latter has only been demonstrated by a limited number of studies [19], and this an important analytic step for quantifying the climate-response relationship for diseases affected by climate change [23]. Finally, these findings support the utility of remote sensing SST data for simple and routine surveillance of potential *Vp* risk.

## Conclusion

Nighttime SST was a significant predictor of *Vp* in BC, Canada. A threshold temperature of 14^o^C indicated increased risk of *Vp* in both oyster meat and human illnesses. This study supports findings from previous studies illustrating the link between SST and *Vp.* Routine surveillance of SST, particularly through readily accessible remote sensing data, could provide early warning of *Vp* risk and help to inform the introduction and cessation of preventative or control measures.

## Abbreviations

BC: British Columbia
BCCDC: British Columbia Centre for Disease Control
MPN: Most probable number
MUR: Multiscale Ultrahigh Resolution
NASA: National Aeronautics and Space Administration
PSU: Practical salinity units
SST: Sea surface temperature
*Vp*: *Vibrio parahaemolyticus*
